# Prevalence of *Chlamydia trachomatis, Ureaplasma urealyticum*, and *Neisseria gonorrhoeae* in Asymptomatic Women from Urban-Peripheral and Rural Populations of Cuenca, Ecuador

**DOI:** 10.3390/idr14050070

**Published:** 2022-08-29

**Authors:** Sebastián Abad, Elizavet Neira, Lourdes Viñansaca, Samuel Escandón, Vivian Alejandra Neira

**Affiliations:** 1 Faculty of Medicine, University of Azuay, Cuenca 010104, Ecuador; 2 Biosciences Department, Faculty of Chemistry, University of Cuenca, Cuenca 010203, Ecuador

**Keywords:** *Chlamydia trachomatis*, flow-through hybridization, *Neisseria gonorrhoeae*, polymerase chain reaction, sexually transmitted disease, *Ureaplasma urealyticum*

## Abstract

Background: Sexually transmitted diseases (STDs) are a serious public health issue due to their high prevalence and a substantial percentage of women being asymptomatic. The present study aimed to determine the prevalence of three STD-causative pathogens in asymptomatic women from Southern Ecuador, with the ultimate purpose of updating the epidemiological data and obtaining a timely diagnosis, which can prevent further complications. Methods: This cross-sectional study included 102 asymptomatic women from Cuenca, Ecuador, who underwent a cervical cytology examination. They met all the inclusion criteria and signed the consent form. Nucleic acids were extracted from each sample, and PCR and flow-through hybridization were performed to detect the pathogens responsible for three STDs. Descriptive and inferential statistics were used to define and describe the study population, obtain the frequency data, and measure central tendencies to determine possible associations among the variables. Results: We found that 49.02% of the participants were infected with at least one of the three microorganisms, with 48.04% and 2.94% carrying *Ureaplasma urealyticum* (UU) and *Chlamydia trachomatis* (CT), respectively. *Neisseria gonorrhoeae* (NG) infection was not observed. Among the participants, 1.96% presented co-infections with CT and UU. Approximately half of the participants presented with asymptomatic infections caused by at least one microorganism. Conclusions: This study demonstrates the importance of conducting regular STD screening programs for high-risk asymptomatic women.

## 1. Introduction

Sexually transmitted diseases (STDs) are mainly transmitted through sexual contact, such as oral, vaginal, or anal intercourse. Nevertheless, some STDs can also be transmitted by other modes, such as hematogenous or vertical (from mother to child during birth) transmission [1]. Therefore, they are a serious public health concern in both developed and developing countries [2].

Currently, the World Health Organization estimates that more than 1 million people acquire STDs daily worldwide. In 2016, approximately 376 million people contracted one of the following four STDs: chlamydia (127 million), gonorrhea (87 million), syphilis (6.3 million), or trichomoniasis (156 million) [2]. In addition, approximately 70% of sexually active women and men present with *Ureaplasma urealyticum* (UU) infection, which causes non-gonococcal urinary tract infection [3]. From an epidemiological perspective, up to 80% of female sexual apparatus infections are asymptomatic or present with mild symptoms [4]. This prevents opportune diagnosis and creates a silent infection reservoir, provoking sustained transmission within a community and the development of future complications (such as infections of the upper urinary tract, infertility, pelvic inflammatory disease, issues in pregnancy, and superinfections) [5].

*Chlamydia trachomatis* (CT) is a Gram-negative bacterium. Its life cycle consists of a metabolically inactive infectious form (elemental bodies) and a non-infectious metabolically active form (reticulate bodies). Cells prone to infection include those of the non-ciliated columnar, cuboidal, and transition epithelium found in the mucous membranes of the urethra, endocervix, endometrium, Fallopian tube, anus, rectum, respiratory tract, and conjunctiva [4]. *Neisseria gonorrhoeae* (NG) is an aerobic, non-motile, Gram-negative coccus with a tendency to pair (diplococci). Infection includes four specific stages: local attachment (through fimbriae, which allow it to adhere to the epithelial cells), invasion, dissemination, and immune evasion. It can grow and multiply in the mucous membranes, including the cervix, uterus, and Fallopian tubes, in women, and in the male urethra [4]. Finally, UU is a relatively small, free-living organism that is difficult to visualize using Gram staining [6]. It remains attached to the epithelial cells of the respiratory or urogenital tract and can disseminate to other locations, causing infection in areas with mucosal disruption [7].

Factors that are consistently associated with a higher probability of infection caused by CT, NG, and UU include multiple sexual partners, belonging to an ethnic minority, low educational and socioeconomic levels, and a history of previous sexually transmitted infections [4,6,8,9,10]. As the number of sexual partners increases, the chances of encountering a partner who is a carrier of an STD also increase [11]. Associations have also been found between age and marital status and the presence of STDs [8,9,10,11]. There is a higher prevalence of chlamydia in people younger than 25 years [9]. It is believed that this may be related to the development of partial immunity [12].

The aim of this study was to update the epidemiological prevalence data of STDs caused by *Chlamydia trachomatis* (CT), *Neisseria gonorrhoeae* (NG), and *Ureaplasma urealyticum* (UU) in asymptomatic women in Cuenca, Ecuador, and to study the relationship of these species with the risk factors frequently associated with their manifestation using a nucleic acid amplification molecular technique, allowing timely diagnosis, treatment, and opportune counseling. To the best of our knowledge, no studies in Ecuador have described the prevalence of STDs in urban versus rural areas.

## 2. Materials and Methods

### 2.1. Patient Recruitment

This was a descriptive, observational, cross-sectional study. The participants included asymptomatic patients visiting the Pablo Jaramillo Humanitarian Foundation in the city of Cuenca, Ecuador, for cervical cytology from May to August 2020. This study was approved by the Ethics Committee of the Universidad San Francisco de Quito (USFQ: P2019-175E) and the Intelligence Direction in Health of the Public Health Ministry (MSPCRI000352-1). In total, 102 participants were recruited based on the following inclusion criteria: fertile women in the age group of 18 to 45 years, sexually active, asymptomatic for STDs (CT, NG, and UU), and not using contraceptive methods regularly. All participants willingly provided written informed consent to participate in this study. Pregnant women, those with an established diagnosis of STDs, women currently being treated for a vaginal infection, and those with a contraindication for performing cervical cytology were excluded from the study. All participants completed a data collection form to specify their sociodemographic characteristics and identify possible risk factors for STDs. The form included questions regarding sociodemographic information, such as age, marital status, ethnic and socioeconomic self-perception, origin (urban or rural), and risk factors such as the onset of sexual intercourse, difficulty in conceiving, miscarriages history, number of sexual partners, highest educational level, previous treatment for vaginal infection, and the use of intrauterine devices.

### 2.2. Sample Collection

Cervical samples were obtained by an expert gynecologist, using a custom-designed cervical brush. The samples were preserved in the cell preservation medium of the Hybribio female sample collection kit (Hybribio, Chaozhou, China) and stored at −2 to 8 °C until further processing. The samples were identified using a numerical code to guarantee patient confidentiality.

### 2.3. Sample Processing

Nucleic acids were isolated from each sample using the Hybribio cell lysis kit (HBCL) (Hybribio, Chaozhou, China), following the manufacturer’s instructions. The STD3 diagnostic kit Hybribio 3-in-1 CT/NG/UU was used for nucleic acid amplification using polymerase chain reaction (PCR), which was carried out on a Veriti thermal cycler (Applied Biosystems, Foster City, CA, USA) under the following conditions: heating of the amplification mixture for 5 min at 37 °C; initial denaturation at 95 °C for 11 min; 40 cycles of: denaturation at 95 °C for 30 s, annealing at 58 °C for 30 s, and elongation at 72 °C for 50 s; and a final elongation at 72 °C for 5 min. The subsequent hybridization was performed using the flow-through hybridization technique using HibriMax (Hybribio Limited, Sheung Wan, Hong Kong). The results were interpreted by direct observation. All tests included positive, negative, and internal controls.

### 2.4. Statistical Analyses

The collected data were entered into a database that allowed percentage calculations of the studied variables. After obtaining the results, descriptive and inferential statistics were used to define and describe the study population, obtain the frequency data, measure the central tendency for the quantitative variables, and determine the associations among the variables.

Data were analyzed using STATA V.14.0 (StataCorp LLC, College Station, TX, USA). Measures of central tendency and dispersion, mean, and standard deviation were calculated for continuous variable data, whereas percentages were calculated for categorical data. Accordingly, continuous variables were analyzed using Student’s *t*-test and the non-parametric Wilcoxon rank-sum test, whereas categorical variables were analyzed using the Chi-square test (χ^2^) or Fisher’s exact test. Multivariate analysis was performed using a logistic regression model for bivariate models according to their relevance to estimate the risk factors related to STDs. In addition, odds ratios (ORs) and 95% confidence intervals (CIs) were calculated. In all tests, a *p*-value of <0.05 was considered statistically significant.

## 3. Results

**Figure 1:**
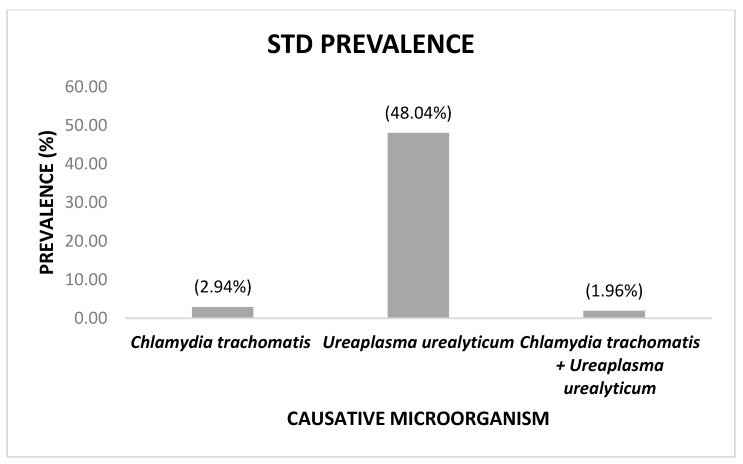
Prevalence of sexually transmitted diseases according to the causative microorganism. Co-infections are included.

In all the cases, sufficient genetic material was obtained from all samples, and the internal control corroborated the reliability of the results. Of the 102 cervical brushing samples from 102 asymptomatic women analyzed, 49.02% of the participants were infected with at least one of the three microorganisms in question. The most prevalent infection was caused by UU alone, followed by CT alone; NG was not detected. Figure 1 shows the prevalence of the microorganisms in STDs.

The mean age of the study participants was 31.54 ± 6.27 years, with no significant difference between the positive and negative cases. Sociodemographic characteristics and risk factors were associated with STD prevalence (Table 1 and Table 2, respectively).

Participants from the urban population contributed to a higher percentage of positive cases than those from rural areas (74% vs. 26%), but the differences were not statistically significant (*p* > 0.05). Differences in the other sociodemographic variables between the positive and negative cases were also statistically insignificant (Table 1).

The risk variables did not show statistically significant associations (Table 2).

Bivariate logistic regression models were conducted, using a dummy as a dependent variable that was equal to 1 if the cases presented with at least one infection. No statistically significant differences were observed. Hence, an adjusted model was not constructed (Table 3).

## 4. Discussion

In this study, a multiplex PCR technique was implemented, followed by flow-through hybridization, to determine the prevalence of three STD-causing pathogens in a simultaneous, fast, and less expensive manner, in comparison with conventional methods. It was found that approximately half of the participants presented with asymptomatic infections caused by at least one of the studied microorganisms.

The diagnosis of STDs has improved, owing to advancements in molecular biology techniques; this has increased the global reporting of statistically relevant epidemiological data. In Ecuador, diagnostic tests are expensive and inaccessible for most health services. Therefore, patients are deprived of timely diagnoses. This situation complicates and hinders the establishment of actual prevalence data. Asymptomatic infections are common and difficult to diagnose. Additionally, the prevalence of mixed infections can be one of the reasons why urethritis and cervicitis are recurrent or persistent [13].

In the current study, it was found that 49.02% of the recruited patients were infected with an STD caused by at least one pathogen. The most prevalent causative agent was UU (48.04%), followed by CT (2.94%), and no cases of NG infection were reported (0%). Additionally, only 1.96% of the participants presented with co-infections with CT and UU.

The prevalence of UU observed in this study is similar to the published global prevalence, where the existence of *Ureaplasma* spp. has been reported in 40–80% of asymptomatic women [6]. The high and variable prevalence of these microorganisms in asymptomatic women raises concerns over their capacity to cause sickness. Nevertheless, there is evidence of their etiological role in different infections and complications in female fertility, [6,7,13] which justifies the need for timely diagnosis. The percentage of UU infections in asymptomatic women observed in this study was similar to that reported by Keane et al. (48%) [14]. However, it was higher than that reported in several European countries. [15,16,17] For example, a study conducted in Russia reported a prevalence of 5.9% among asymptomatic women [18]. Of note, UU colonization is associated with ethnicity [6,14]. This could explain the higher prevalence of UU among women from the American continent compared with those from European countries. The study findings reflect regional variability; however, epidemiological data for these infections are lacking, especially in Latin America. Therefore, it is necessary to perform large-scale studies to confirm possible differences.

Currently, the screening and treatment of STDs caused by UU infections and other non-traditional STDs are controversial. This is because most women with UU colonization do not develop genital tract infections. Therefore, the European guidelines do not recommend the treatment of UU infections for asymptomatic women. Screening for UU can cause overdiagnosis and unnecessary treatment, along with socioeconomic implications and bacterial resistance to pathogens [19]. Nevertheless, a meta-analysis conducted in Iran suggested a high prevalence of infertility in women colonized by UU [20]. Thus, we believe that although it is controversial, treating an asymptomatic infection can influence the prevalence of infertility. Currently, there is a lack of evidence regarding effective treatment regimens. It is necessary to differentiate between colonization and infection, and only individuals with a high UU load should be considered for treatment [16].

CT infections cause a highly prevalent STD that remains asymptomatic in most infected patients (approximately 61%) [21]. Consequently, it becomes a continuous source of transmission and multiple complications during the female reproductive life course [5]. These consequences are the main reasons why CT infections are considered the most expensive non-viral STD in countries such as the United States [22]. Thus, screening sexually active women can decrease complication rates and reduce public health expenses. A meta-analysis by Huai et al. reported that the global prevalence of CT infections in the general population is 2.9%, which is consistent with the findings of the present study (2.94%) [21]. Similarly, a study conducted in Brazil among asymptomatic women reported CT infections in 2.83% of the cases [23]. A lower prevalence has been observed in European and Asian countries. A study conducted in Turin in asymptomatic women of fertile age found a CT prevalence of 1.4% [17]. Additionally, a South Korean study in clinically healthy women found a CT prevalence of 0.5% [24]. The Pan-American Health Organization has reported the highest prevalence (worldwide) of chlamydia in the region of the Americas, which is consistent with our findings [25]. Few studies have elucidated regional variations in prevalence; however, several factors (sociocultural, economic, sexual practices, gender inequity, circumcision, and access to STD screening) can influence the data obtained [5,21,26].

Co-infections with CT and other sexually transmitted microorganisms are frequent in high-risk women [13]. For example, a study conducted among women who visited an STD clinic in Estonia revealed frequent co-infections with CT and UU. The authors concluded that if a patient presents with CT infection, the person has a 2.6% risk of being co-infected with UU [27]. Although we found co-infection with CT and UU in only 1.96% of the cases, two of the three positive asymptomatic women with CT infection were co-infected with UU. Few epidemiological studies report CT and UU co-infection in asymptomatic women [16,28]. Interestingly, *Mycoplasma* may play a role in the establishment of chlamydial persistence by depleting nutrients and host cell biosynthetic precursors [17,29].

The global prevalence of gonorrhea in women is 0.9% [30]. In the current study, the prevalence of NG in asymptomatic women was 0%. The absence of NG infection in the present study corresponds with the results of several studies performed on asymptomatic women [23,31]. However, about 50% of the women infected with NG are asymptomatic [23]. The known resistance of NG to multiple antibiotics has made it a multidrug-resistant organism of global concern [32]. Therefore, it is important to address the prevention and early treatment of this infection.

Although statistically significant associations were not found in the present study, the prevalence of STDs in the study participants was higher among those from urban zones than those from the rural zones (74% vs. 26%). These differences may be because the prevalence of genital microorganisms is related to regional differences (such as sexual practices, access to health, and economic inequality) [33]. The statistical insignificance of the data could be because the Latin American rural world has undergone transformations related to modernization and globalization. The development of new technological tools, mainly the internet, has increased accessibility of information to a considerable population, which has allowed the homogenization of fashion, customs, and behaviors; urban and rural boundaries have become obscure [34]. The organization of urban industrialized societies entails a lifestyle that contributes to the high prevalence of STDs. This could be the result of long working hours, and geographic and social mobility that foments casual sexual relations, as well as other social, cultural, economic, and educational factors [35].

Meanwhile, the association between the risk factors and STD prevalence was not statistically significant. However, the risk of STD increases with an increase in the number of sexual partners [4,6,14]. Statistical insignificance could be caused by the participants shying away from answering questions related to sexual background accurately.

Finally, some of the results can be considered random; therefore, further studies in different environments are necessary to identify possible associations among the described STD variables.

The most significant limitation of the present study was the limited geographic area, which included only the center where the samples were collected. Women who visited the clinic were mainly from the Ecuadorian South; therefore, the data do not represent national STD prevalence. The study should further be expanded to other regions around Ecuador to obtain a better estimate of the national prevalence and its association with the proposed risk factors.

In conclusion, the study findings are a reliable estimate (similar to results of other studies globally) of STD prevalence in the area included in our study. Owing to the high percentage of STD-positive asymptomatic women, the study recommends screening programs in high-risk women to prevent complications and further transmission of STDs.

## Figures and Tables

**Table 1:** The association of sociodemographic variables with positive or negative results obtained for sexually transmitted diseases (STDs) caused by *Chlamydia trachomatis*, *Neisseria gonorrhoeae*, and *Ureaplasma urealyticum*.

Sociodemographic Variables	Total (*n* = 102)	Positive (*n* = 50)	Negative (*n* = 52)	*p*-Value
**Age (years) M (SD)**	31.54 (6.27)	31.08 (6.08)	31.98 (6.47)	0.584 *
**Residence *n* (%)**				0.747 ^‡^
**Urban**	74 (72.55)	37 (74)	37 (71.15)	
**Rural**	28(27.45)	13 (26)	15 (28.85)	
**SES *n* (%)**				0.313 ^‡^
**Medium**	74 (72.55)	34 (68)	40 (76.92)	
**Low**	28 (27.45)	16 (32)	12 (23.08)	
**Highest educational level *n* (%)**				0.879 ^†^
**Primary school**	21 (20.59)	9 (18)	12 (23.08)	
**High school**	37 (36.27)	19 (38)	18 (34.62)	
**University graduate**	41 (40.20)	21 (42)	20 (38.46)	
**Postgraduate**	3 (2.94)	1 (2)	2 (3.85)	
**Marital status *n* (%)**				0.596 ^†^
**Single**	33 (32.35)	19 (38)	14 (26.92)	
**In a relationship**	12 (11.76)	5 (10)	7 (13.46)	
**Married**	48 (47.06)	21 (42)	27 (51.92)	
**Divorced**	9 (8.82)	5 (10)	4 (7.69)	
**Ethnicity *n* (%)**				0.485 ^†^
**Mestizo**	99 (97.06)	48 (96)	51 (98.08)	
**White**	3 (2.94)	2 (4)	1 (1.92)	

*n* = sample size. SES = socioeconomic status. M (SD): Mean and standard deviation. * *p*-value was calculated using the parametric Student *t*-test. ^†^ *p*-value was calculated using the non-parametric Pearson chi-square test. ^‡^ *p*-value was calculated using the non-parametric Fisher exact test.

**Table 2:** Association of risk variables with positive or negative *C. trachomatis*, *N. gonorrhoeae*, *and U. urealyticum* results.

Risk Variables	Total (*n* = 102)	Positive (*n* = 50)	Negative (*n* = 52)	*p* Value
**Sexual partners ^¥^ M (SD)**	2.55 (2.1)	2.64 (2.28)	2.47 (1.91)	0.626 ^‣^
**Onset of sexual intercourse (age) M (SD)**	17.93 (3.52)	17.74 (3.12)	18.12 (3.89)	0.635 ^‣^
**Miscarriages M (SD)**	0.29 (0.57)	0.3 (0.50)	0.29 (0.64)	0.514 ^‣^
**Difficulties in conception *n* (%)**				0.893 ^‡^
**No difficulty**	77 (75.49)	38 (76)	39 (75)	
**Did not attempt conception**	11 (10.78)	6 (12)	5 (9.62)	
**Has had difficulty**	14 (13.73)	6 (12)	8 (15.38)	
**Miscarriage diagnosis** ***n* (%)**				0.422 ^‡^
**Yes**	25 (24.51)	14 (28)	11 (21.15)	
**No**	77 (75.49)	36 (72)	41 (78.85)	
**Number of miscarriages *n* (%)**				0.430 ^‡^
**0**	77 (75.49)	36 (72)	41 (78.85)	
**1**	21 (20.59)	13 (26)	8 (15.38)	
**2**	3 (2.94)	1 (2)	2 (3.85)	
**3**	1 (0.98)	0 (0)	1 (1.92)	
**Treated for vaginal infection *n* (%)**				0.907 ^†^
**Yes**	77 (75.49)	38 (76)	39 (75)	
**No**	25 (24.51)	12 (24)	13 (25)	
**Copper T or IUD *n* (%)**				0.732 ^†^
**Yes**	25 (24.51)	13 (26)	12 (23.08)	
**No**	77 (75.49)	37 (74)	40 (76.92)	

*n* = sample size. M (SD): mean and standard deviation. ‣ *p*-value was calculated using the non-parametric Wilcoxon rank-sum test. ^‡^ *p*-value was calculated using the non-parametric Fisher exact test. ^†^ *p*-value was calculated using the non-parametric Pearson chi-square test. IUD: Intrauterine device. ^¥^ *n* = 101, as one participant did not answer the corresponding question.

**Table 3:** Bivariate logistic regression (*n* = 102).

Bivariate Models					
Independent Variables	OR *	SE ^†^	Confidence Interval		*p* Value
			Lower Limit	Upper Limit	
**Age (years)**	0.98	0.03	0.92	1.04	0.467
**Sexual partners**	1.04	0.10	0.86	1.26	0.684
**Onset of sexual intercourse**	0.97	0.06	0.87	1.08	0.590
**Abortion history**	1.45	0.67	0.58	3.59	0.423
**Residence (rural)**	0.87	0.38	0.36	2.07	0.748
**SES (low)**	1.57	0.70	0.65	3.77	0.314
**Education level (Primary school)**					
**High school**	1.41	0.77	0.48	4.14	0.534
**University graduate**	1.4	0.76	0.49	4.04	0.534
**Postgraduate**	0.67	0.87	0.05	8.55	0.755
**Marital status (Single)**					
**In a relationship**	0.53	0.36	0.14	2.01	0.348
**Married**	0.57	0.26	0.23	1.40	0.223
**Divorced**	0.92	0.70	0.21	4.07	0.914
**Ethnicity (White)**	2.13	2.64	0.19	24.20	0.544
**Difficulty in conception (Yes)**	0.75	0.44	0.24	2.34	0.620
**Miscarriage diagnosis (Yes)**	1.45	0.67	0.58	3.59	0.423
**Vaginal infection (Yes)**	1.06	0.49	0.43	2.60	0.907
**IUD (Yes)**	1.17	0.54	0.47	2.89	0.732

^†^ SE: standard error. * OR: odds ratio. IUD: intrauterine devices.

## Data Availability

Not applicable.
